# Scavenging of Organic Pollutant and Fuel Generation through Cost-Effective and Abundantly Accessible Rust: A Theoretical Support with DFT Simulations

**DOI:** 10.3390/ma16010142

**Published:** 2022-12-23

**Authors:** Nisar Khan, Tamanna Gul, Idrees Khan, Eman A. Alabbad, Shahid Ali, Khalid Saeed, Ibrahim Khan

**Affiliations:** 1 Department of Chemistry, Bacha Khan University, Khyber Pakhtunkhwa, Charsadda 24540, Pakistan; 2 School of Chemistry and Chemical Engineering, Northwestern Polytechnical University, Xi’an 710129, China; 3 Department of Chemistry, College of Science, Imam Abdulrahman Bin Faisal University, P.O. Box 1980, Dammam 31441, Saudi Arabia; 4 Interdisciplinary Research Center for Hydrogen and Energy Storage, King Fahd University of Petroleum and Minerals, Dhahran 31261, Saudi Arabia; 5 School of Chemical Engineering and Materials Science, Chung-Ang University, 84 Heukseok-ro, Seoul 06974, Republic of Korea

**Keywords:** calcination, rust, photocatalyst, photodegradation, methylene blue, water splitting

## Abstract

Waste management and energy generation are the foremost concerns due to their direct relationship with biological species and the environment. Herein, we report the utilization of iron rust (inorganic pollutant) as a photocatalyst for the photodegradation of methylene blue (MB) dye (organic pollutant) under visible light (economic) and water oxidation (energy generation). Iron rust was collected from metallic pipes and calcined in the furnace at 700 °C for 3 h to remove the moisture/volatile content. The uncalcined and calcined rust NPs are characterized through scanning electron microscopy (SEM), energy dispersive spectroscopy (EDS), Fourier-transform infrared (FTIR) analysis, X-ray Diffraction (XRD), and thermogravimetric analysis (TGA). The morphological study illustrated that the shape of uncalcined and calcined iron rust is spongy, porous, and agglomerated. The XRD and DLS particle sizes are in a few hundred nanometers range. The photodegradation (PD) investigation shows that calcined rust NPs are potent for the PD of modeled MB, and the degradation efficiency was about 94% in a very short time of 11 min. The photoelectrochemical (PEC) measurements revealed that calcined rust NPs are more active than uncalcined rust under simulated 1 SUN illumination with the respective photocurrent densities of ~0.40 and ~0.32 mA/cm^2^. The density functional theory simulations show the chemisorption of dye molecules over the catalyst surface, which evinces the high catalytic activity of the catalyst. These results demonstrate that cheaper and abundantly available rust can be useful for environmental and energy applications.

## 1. Introduction

Photocatalysis is a felicitous process and the most appropriate technology for the degradation of pollutants, since it is cheap, reliable, efficient, and environmentally friendly [1]. Various approaches are adopted to efficiently utilize this technique for energy and environmental applications, such as pollutants degradation, CO_2_ reduction, etc. These approaches cover from sophisticated device fabrication to wide material utilization [2,3,4,5]. Photocatalysts are a large class of materials comprised of organic conjugated polymers [6], supramolecular complexes [7], and, most important, semiconducting materials [8,9,10,11,12,13,14,15,16,17,18,19,20,21,22], etc. Semiconducting materials are preferred photocatalysts due to their good efficiency in utilizing solar energy within the visible region to remove organic pollutants and generate green hydrogen from photoelectrochemical (PEC) water splitting [23,24,25,26,27]. Among the semiconducting materials, Fe_2_O_3_ is considered a promising photocatalyst due to its excellent stability, availability, cost, low bandgap (2.3 eV), and good harvesting capability of visible light. Additionally, low toxicity, environmental compatibility, and recyclability are the other attractive features of Fe_2_O_3_ [28,29,30]. Fe_2_O_3_ photocatalytic materials are utilized in many advanced fields, such as adsorption [31], solar cells [32], water splitting [33,34,35], and dye degradation [36,37,38], and have shown exceptional outcomes. However, producing photocatalytic Fe_2_O_3_ nanoparticles (NPs) through conventional methods, such as hydrothermal, sol–gel, etc., requires more resources, time, energy, and human efforts, making the product expensive and limited, which is a significant barrier from a commercial perspective. Moreover, hazardous chemicals/precursors are employed in some conventional syntheses that are environmentally unfit [39]. Hence, developing an alternative, safe, and economical approach is always desirable to synthesize much-needed materials for various industrial applications.

In this work, we devised a facile, safe, and economically feasible extracting route to obtain a bulk amount of Fe_2_O_3_ NPs from iron rust (Fe_2_O_3._nH_2_O ). A simple temperature and cleaning treatment of rust results in Fe_2_O_3_ NPs’ dominant product. The dominant Fe_2_O_3_ NP-containing product (calcined rust) is utilized as a photocatalyst for the photodegradation (PD) of a model methylene blue (MB) dye in an aqueous medium and photoelectrochemical (PEC) water splitting. The photocatalytic degradation of MB dye by calcined rust NPs was also performed theoretically by applying DFT calculations that were consistent with the experimental results. Limited studies have reported on recycling and converting iron rust into catalysts, such as the g-C_3_N_4_-Fe_2_O_3_ photocatalyst [40], LaFeO_3_ perovskite-type catalysts [41], and Fe_2_O_3_ from toner powder [42]. These methodologies mostly prepared composite-type catalysts with difficult preparation conditions, ultimately increasing the process costs. Hence, the present study is economical in terms of photocatalyst preparation and feasible for harvesting visible light instead of hazardous and expensive UV light.

## 2. Materials and Methods

### 2.1. Materials

Iron rust was collected from water-transferring pipes in the locality of Lower Dir, KP, Pakistan. Nitric acid (HNO_3_) and sodium hydroxide (NaOH) were obtained from Sigma Aldrich,, St. Louis, USA. Methylene blue dye was purchased from Scharlau Chemicals, Turkey.

### 2.2. Preparation of Photocatalyst

The collected iron rust was first washed with distilled H_2_O to remove any attached impurities and then dried in an oven at 130 °C for 5 h. The dried rust was calcined at 700 °C for 3 h to remove the adhered moisture content. The high temperature removes the attached water molecules from the iron rust (Fe_2_O_3_·nH_2_O) and converts them into a predominantly Fe_2_O_3_ NP-containing product (henceforward called calcined rust NPs), as can be seen in Equation 1. After calcination, the sample was cooled in a desiccator and then packed in order to avoid moistening.

Fe_2_O_3_·nH_2_O ⟶ Fe_2_O_3_ + nH_2_O (1)

Due to the complex nature of rust, it contained unresolved impurities, which are expected to exploit the photocatalytic activity of these catalysts. 

### 2.3. Photodegradation Study of Methylene Blue Dye

The calcined rust NPs were utilized as photocatalysts for the PD of MB dye under visible light in an aqueous solution as a function of different operational parameters, e.g., irradiation time, photocatalyst reusability, catalyst amount, and initial dye concentration. In the time study, 0.02g (2g/L) of the photocatalyst was added to a 10 mL solution of MB dye (15 ppm) and sonicated for 2 min for complete dispersion of calcined rust NPs. The sample was then kept in the dark to attain adsorption–desorption equilibrium. The sample was then kept under standard light with constant and continuous stirring for different irradiation times (1, 3, 5, 7, 9, and 11 min). After the reaction time completion, the photocatalyst was separated through centrifugation (12,000 rpm for 10 min).

The %degradation of MB dye is calculated from the following formulas:

(2) $$\mathrm{Degradation}\;(\%)=\left(\frac{C_{0}-C}{C_{0}}\right)\times 100$$

(3) $$\mathrm{Degradation}\;(\%)=\left(\frac{A_{0}-A}{A_{0}}\right)\times 100$$

where *C*_0_ represents the initial concentration of dye, *C* stands for dye concentration after the reaction, *A*_0_ symbolizes initial absorbance, and *A* shows the absorbance of dye after the reaction.

### 2.4. DFT Simulation Details

All the computations were performed with DFT in DMOl3 code [43,44] using a material studio graphical interface. The level of theory used for all quantum chemical calculations is the Perdew–Burke–Ernzerhof (PBE) [45] formulation of the generalized gradient approximation (GGA). The DFT+D2 method (Grimme’s scheme) was applied for a long-range dispersion correction in intermolecular interactions [46,47]. The double-numerical plus polarization (DNP) basis set was employed for calculations that are comparable in obtaining good results to those of Pople’s 6-31G(d,p) basis set. The Fermi-smearing was set to 0.005 Ha, and a real space cutoff of 4.6 Å was utilized in the calculations. The geometries of all systems (monomers and complexes) were optimized at a convergence tolerance of 10^−5^ Ha, 0.001 Ha/Å, and 0.005 Å for the energy, force, and displacement, respectively. For each adsorbate, the energy of adsorption (Ead) was gained as:

E_ad_ = E_complex_ − (E_M1_ + E_M2_) (4)

where E_complex_ corresponds to the total energy of the complex system (dye adsorbed over the Fe_2_O_3_), while E_dye_ and E_Fe2O3_ represent the total energies of the isolated monomers dye and Fe2O3, respectively. The frequency calculations at temperature 298 K and pressure 1.00 atm were performed using the same level of theory to confirm the stability of all optimized structures. The absence of an imaginary frequency proves that all the structures are at their potential minima.

### 2.5. Photoelectrode Fabrication and Photoelectrochemical Measurements

PEC water splitting of uncalcined and calcined rust NPs was performed in a three-electrode PEC cell containing 0.5 M Na_2_SO_4_ (Sigma Aldrich, St. Louis, MI, USA) electrolyte (pH 7.2). The uncalcined and calcined rust NPs deposited FTOs were used as the working electrodes and were controlled by an Autolab potentiostat. Platinum (Pt) and saturated calomel electrodes (SCE) served as the counter and reference electrodes. To prepare the working electrode, we dissolved 2 mg of the photocatalyst in 2mL DI water, which was sonicated for 30 min, followed by adding one drop of 5% Nafion (as a binder). The dispersed solution was dropped and cast over 0.25 mm^2^ conducting glass (fluorinated tin oxide). All PEC measurements were recorded using NOVA software installed on a data collecting device under the controlled chopping of dark and light. A solar simulator (Oriel Sol-3A Newport) provided artificial solar light irradiation, and the power was calibrated using a silicon diode solar cell (Oriel-diode) and fixed at 100 mW/cm^2^, which is equivalent to 1 SUN. The solar simulator was also equipped with AM-1.5 G and UV cutoff (λ > 420 nm) filters.

### 2.6. Morphological, Structural, and Optical Characterizations 

The uncalcined and calcined rust NPs were characterized through Fe-SEM and EDX (LA-6490, JEOL Japan, in the energy range of 0–20 keV). Each sample was prepared from the dry sample in ethanol, followed by sonication for 30 minutes. The FTIR analysis was carried out via FTIR (Nicolet Nexus 470, Thermo Electron Co., Waltham, MA, USA). The photodegradation study was performed by a UV–Visible spectrophotometer (Model = Shimadzu 1800, Japan). Dynamic light scattering (particle size distribution and zeta potential) was calculated. X-ray diffraction (XRD) was carried out using the Rigaku Miniflex II Desktop X-ray diffractometer for a 2θ range of 5–80°, sampling step size of 0.03°, and 3.00 scan speed. The particle size of the synthesized materials was measured by Zetasizer nano Series dynamic light scattering (DLS = ZEN3600, Malvern, UK). All the samples were sonicated before each run for 15 minutes by a probe sonicator (UP400St, Hielscher, Teltow, Germany) in ethanol. The surface area was analyzed by a Micromeritics ASAP 2020 BET analyzer. Initially, the samples were degassed at 180 °C for 4 h under a vacuum to eliminate impurities prior to N_2_ physisorption measurements. The TGA analysis was performed using a Mettler Toledo analyzer (TGA/SDTA-851e, Switzerland) under a nitrogen environment, with temperatures from 25 to 800 °C at a scan rate of 10 °C/min, to determine the thermal properties of the calcined and uncalcined samples.

## 3. Results and Discussions

### 3.1. Morphological, Elemental, and Mapping Analysis

**Figure 1:**
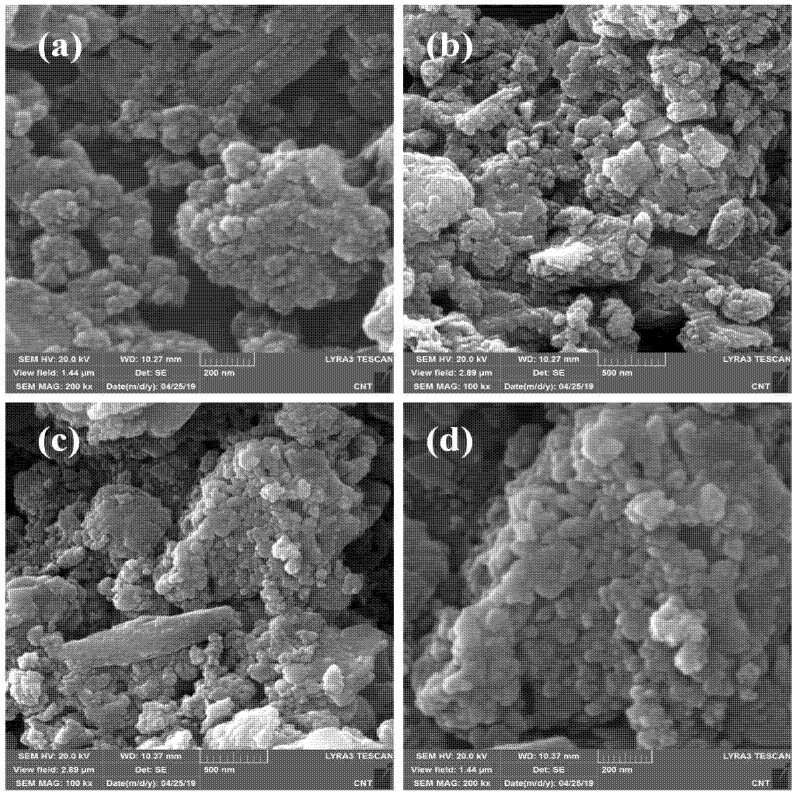
SEM images of (**a**,**b**) uncalcined iron rust NPs and (**c**,**d**) calcined rust NPs.

A SEM analysis is a powerful tool for studying the surface morphological features of the photocatalyst. Figure 1a–d represents the lower and higher magnification SEM micrographs of the as-collected uncalcined and calcined iron rust, respectively. After comparing the morphologies, it is clear that both samples show significant agglomerations with highly irregular morphologies. Moreover, the SEM micrographs indicate that the individual particle sizes in both cases are in the nanometer range, agglomerated into large blocks of irregular particles. We assume other parameters could promote the photo- and photoelectrocatalytic performance of calcined rust NPs compared to uncalcined rust under similar conditions. Therefore, we characterized these samples further with XRD, BET, and TGA to confirm those parameters contributing to the enhanced catalytic performance.

**Figure 2:**
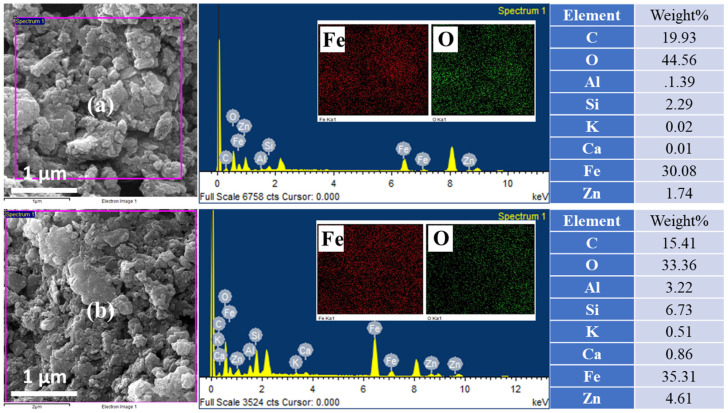
EDX and elemental mapping of (**a**) uncalcined iron rust NPs (top) and (**b**) calcined iron rust NPs (bottom).

**Figure 3:**
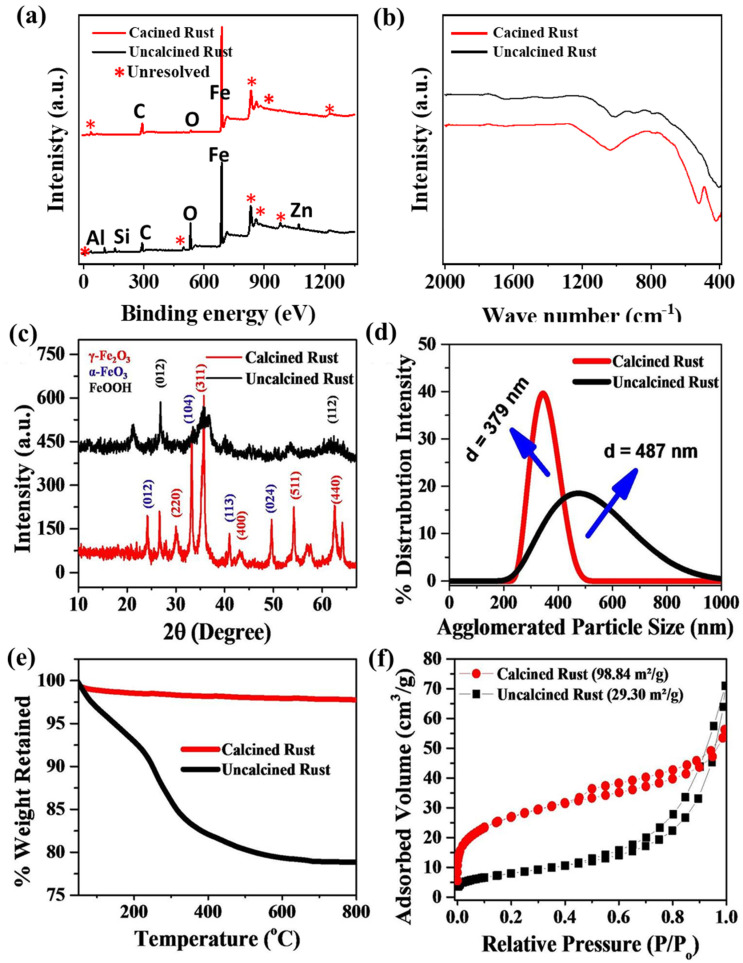
Chemical and structural analyses of uncalcined and calcined rust NPs. (**a**) XPS survey, (**b**) FTIR, (**c**) XRD patterns, (**d**) particle size distribution, (**e**) thermogravimetric analysis, and (**f**) BET surface area analysis of uncalcined and calcined iron rust.

Figure 2 illustrates the elemental analysis and mapping study of uncalcined and calcined rust. Figure 2a shows the EDX spectra of uncalcined rust with %elemental composition in tabular form. The representing peaks for Fe, C, Zn, Si, Al, and O are observed with respective % compositions of each element. As expected, the % compositions indicate that Fe and O are present in higher concentrations than Zn, Si, and Al, which exist in minuscule quantities. The presence of Zn, Si, and Al elements can be justified, as the rust was collected from a steel source that contains these metals in proportions (to enhance their strength). Figure 2b–d present the Fe, O, and Si mapping results. The mapping study indicates the presence of Si due to earthy impurities, as rust is obtained from groundwater pipes. Figure 2e demonstrates the EDX spectra and % elemental composition (tabular form) of calcined iron rust, displaying peaks for C, Zn, K, Si, Ca, Al, and O. The percentages of Fe and O were found to be higher than other elements. The results are in agreement with the XPS survey in Figure 3a. 

Furthermore, the percentage of oxygen in the case of calcined rust NPs is lower than the uncalcined rust NPs, which might be due to the elimination of any attached water and other volatile matter. The quantity of C is also reduced after calcination due to its oxidation to CO_2_. Moreover, in the case of calcined rust, we observed K and Ca in trace amounts, which could possibly be added from the ceramics where we calcined the rust at a high temperature, i.e., 700 °C, and the mapping results are shown in Figure 2f–h. The mapping images revealed that all the elements were uniformly dispersed throughout the whole material. It is essential to state that we deliberately did not remove the various elemental impurities to see their overall effect on the photocatalysis and avoid any additional cost for purifying these materials at a large scale. 

### 3.2. Structural Analysis of Uncalcined and Calcined Rust NPs

The XPS survey analysis was performed to understand the chemical composition of the rust. As expected, the survey has shown a mixture of components in the uncalcined rust. However, in the case of calcined rust, the impurities are minimized, yet there are some unresolved impurities, indicated as * in the spectra in Figure 3a. FTIR spectroscopy is known for its high sensitivity, especially in detecting inorganic and organic species with low contents. Figure 3b shows the FTIR spectra of uncalcined and calcined rust representing various peaks in different regions. The uncalcined rust NPs show peaks at about 1033 cm^−1^, possibly due to the lepidocrocite (ferric mineral) [48]. The peaks at about 903 and 799 cm^−1^ might be due to the goethite (hydrated iron oxide) [49]. The absorption peak in the absorption band of uncalcined rust at 1630 cm^−1^ is the characteristic peak of -OH, indicating a large amount of crystal water in the corrosion products [50]. This peak almost disappears in the calcined rust NPs due to high-temperature calcination. Similarly, the calcined iron rust NPs also gives peaks at about 1033, 903, and 799 cm^−1^, which might be due to the lepidocrocite and goethite, respectively. The spectrum of calcined iron rust also presented prominent peaks at about 526 and 436 cm^−1^ due to the stretching vibration of Fe-O [51].

The uncalcined and calcined chemical compositions, phase purity, and crystallinity were identified via XRD analysis. The XRD patterns of the uncalcined and calcined iron rust are shown in Figure 3c. The XRD pattern of the uncalcined rust NPs indicates a mixture of FeOOH and iron oxide (γ-Fe_2_O_3_). On the other hand, the calcined rust NPs show diffraction peaks balanced at 30.18°, 57.28°, and 62.94° corresponding to the (220), (511), and (440) planes of γ-Fe_2_O_3_ iron (JCPDS file no. 75-1594). The peaks located at 24.26°, 33.17°, 40.82°, and 49.57° match well with the (012), (104), (113), and (024) planes of α-Fe_2_O_3_ (JCPDS file no. 33-0664). This means that calcined rust NPs are a combination of γ-Fe_2_O_3_ and α-Fe_2_O_3_ iron. The quantification of XRD patterns indicates that the sample contains ~75% α-Fe_2_O_3_ and ~25% γ-Fe_2_O_3._ The crystallite particle can be given using the Debye–Scherrer equation [52,53]. However, in the case of agglomerated particles and high roughness, it causes the particle size to be magnified in SEM and, therefore, can be misleading if compared [54].

The calcination temperature efficiently transforms thermodynamically unstable iron oxides to the Fe_2_O_3_ phase by losing water molecules [30,55,56]. To further see the effect of calcination on the particle size, we also performed the DLS analysis. The DLS particle size distribution of uncalcined and calcined rust NPs is represented in Figure 3d. The uncalcined rust NPs have a broad peak representing their agglomeration, and the average particle size of uncalcined rust was found to be as high as ~487 nm. Comparatively, the calcine rust sample demonstrates a significantly sharp peak area, which indicates a sharp particle distribution. The calculated average particle size of the calcined sample is measured to be ~379 nm. These DLS results indicate that the calcination reduced the particle size significantly, which can add incremental characteristics to the photocatalytic behavior of rust. Dehydration and phase conversion due to calcination at high temperatures cause a decrease in attractive forces. Thus, the calcined rust NPs are dispersed and display smaller particle sizes than uncalcined rust. The thermogravimetric analysis (TGA) measurements of both samples were carried out to find their thermal stability, and the results are represented in Figure 3e. The isotherms indicate that the uncalcined iron rust lost about 21% weight at a maximum temperature of 800 °C.

The attached and adsorbed water is removed at above 120 °C. The maximum weight loss occurs between 200 and 300 °. However, no significant weight loss is observed above 500 °C. The calcined rust NPs only lost 2% mass during the thermal analysis, which shows the thermostable nature of the calcined samples, as their calcination is performed at a very high temperature of 700 °C. Finally, the effect of calcination over the surface area is also performed through the Brunauer–Emmett–Teller (BET) adsorption method with nitrogen gas. It can be seen in Figure 3f that both isotherms are of type IV. The calcination increased the specific surface area (SSA) of calcined rust NPs to 98.84 m^2^/g, which was 29.30 for an uncalcined sample. The three-times enhancement in the SSA indicated that the crystallization and phase transformation tool took place after calcination, reducing the particle size, as indicated by the XRD and DLS results. 

### 3.3. Photodegradation of Methylene Blue

**Figure 4:**
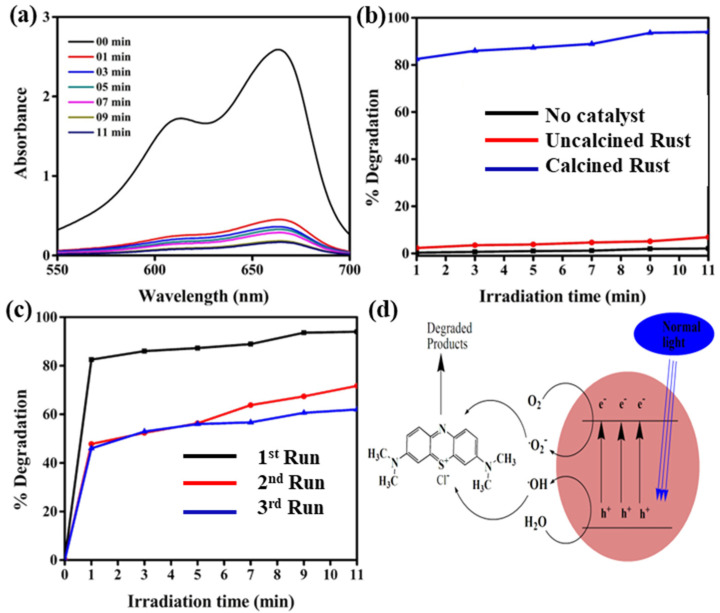
(**a**) UV–Vis spectra of MB photodegraded by calcined rust NPs in aqueous medium under visible light; (**b**) %degradation comparison of MB dye photodegraded by uncalcined rust, calcined rust NPs, and without photocatalysts; and (**c**) reusability of calcined rust NPs. (**d**) Proposed mechanism for the photodegradation of MB, adopted from [59].

The obtained Fe_2_O_3_·nH_2_O (uncalcined) and predominantly Fe_2_O_3_ (calcined) NPs were utilized as photocatalysts for the PD of MB dye in an aqueous medium under visible light. Figure 4a represents the UV–Vis spectra of MB dye before and after different visible light reaction times in the presence of calcined rust NPs, displaying that dye degradation increases with the increasing irradiation time. Figure 4b shows the %degradation of MB dye in the presence of uncalcined, calcined rust NPs, and without photocatalysts (photolysis). No considerable degradation was observed without photocatalyst and uncalcined rust NPs, while calcined rust NPs quickly degraded MB dye. The uncalcined rust has little adsorption capability and thus displays less photocatalytic efficiency. The increased photocatalytic efficiency of calcined rust NPs to uncalcined rust is due to its high-temperature calcination, which makes the materials porous and ultimately adsorb the dye efficiently, followed by drastic PD. It was reported that calcination enhances catalyst activity [57]. Calcined rust NPs obtained from calcined rust are superior in terms of synthesis, low preparation cost, and photocatalytic activity. A comparison of our study with the photocatalytic activities of pure Fe_2_O_3_ reported in the literature is consolidated in Table 1. The table clearly demonstrates that our economical photocatalysts are very reactive in the photodegradation of MB dye compared to the provided list of photocatalysts.

The result shows that calcined rust NPs rapidly degraded 82.5% dye within 1 min, and then, a small increase was observed in the efficiency of PD, and finally, degraded about 94% dye within 11 min. Initially, the degradation efficiency is much faster and slows down upon increasing the irradiation time. This is because, in the beginning, the formation of ^•^OH radicals is faster, and there is more availability of the active site for dye adsorption. After a particular reaction time, the remaining active site is not easy to fill because of the repulsive force between the molecules on the surface with the bulk phase, and thus, after some time, the %degradation tends to be constant [58]. The sustainability of calcined rust NPs is evaluated by utilizing recovered and rerecovered calcined rust NPs under the same experimental conditions. The photocatalyst was recovered by washing with distilled water and oven drying at 100 °C. Figure 4c compares the %degradation of MB dye PD by fresh, recovered, and rerecovered calcined rust NPs. The results revealed that the fresh calcined rust NPs (first run) degraded about 94% MB dye within 11 min, while the recovered (second run) and rerecovered (third) run degraded about 71.7% and 62% dye, respectively, within the same irradiation time. The decrease observed in the photocatalytic activity of the recycled photocatalysts might be due to the blockage of active surface sites by the deposition of photo-insensitive hydroxides [59].

The photodegradation of MB dye occurs as visible light adsorbed by calcined rust NPs that results in the excitation of electrons (e^−^) from the valence band (VB) to the conduction band (CB), creating positively charged holes (h^+^) in the valence band. The resonance effect between the free electrons and photons on the metal surface runs through the entire light region. The hole in the VB reacts with H_2_O molecules and produces hydroxyl radicals (^•^OH), while the e^−^ in the CB reacts with an oxygen molecule and produces superoxide anion radical (^•^O_2_^−^). These generated radicals are highly reactive and degraded MB dye molecules into intermediate products and, finally, into more unaffected species such as CO_2_ and H_2_O, as shown in Figure 4d. The possible reaction steps in this mechanism are summarized in the following equations [59,60].

NPs → NPs (e^−^ + h+) (5)

O_2_ + e^−^ → ^•^O_2_^−^ (6)

H_2_O/OH^−^ + h^+^ → ^•^OH (7)

Dye + ^•^OH + ^•^O_2_^−^→ degraded products (8)

**Table 1:** Comparison of the photodegradation of MB dye by our photocatalysts and Fe2O3-based photocatalysts in the literature.

S.No	Photocatalysts (Synthesis)	%Degradation and Reaction Conditions	Ref
1.	Fe_2_O_3_ (hematite) NPs (combustion)	65.67% in 180 min under UV	[61]
2.	α-Fe_2_O_3_ nanospindles (hydrothermal)	78% in 360 min under UV	[62]
3.	α-Fe_2_O_3_ NPs (solvothermal)	46% in 60 min under xenon	[63]
4.	Fe_2_O_3_ NPs (green synthesis)	94% in 110 min under sunlight	[64]
5.	Fe_2_O_3_ NPs (combustion)	63.64 % in 120 min under UV	[65]
6.	Calcined rust NPs containing Fe_2_O_3_ NPs predominantly (calcination of rust)	94% in 11 min under UV-light	This work

**Figure 5:**
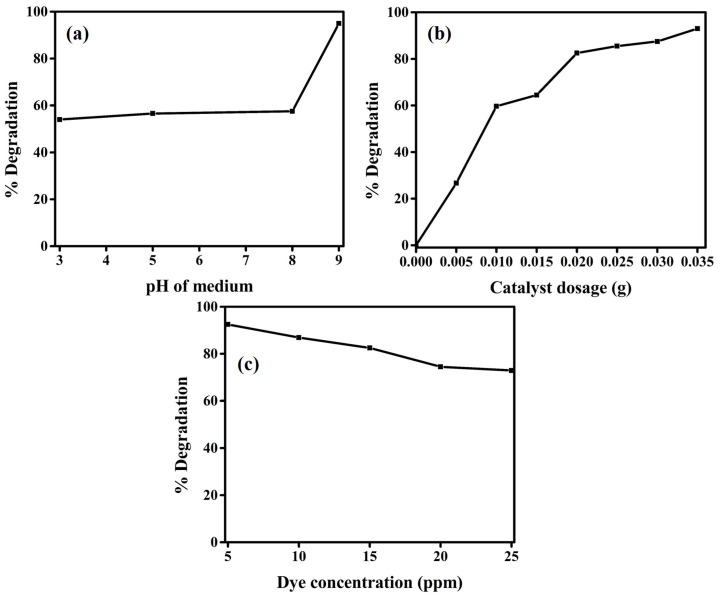
(**a**) Effect of pH of the medium on the MB PD (**b**) effect of calcined rust NPs dosage on the MB PD (**c**) effect of the initial concentration of MB on the PD.

As observed, that calcined rust NPs is much more active than uncalcined rust. Therefore, in other parameter studies, only calcined rust NPs are applied. The effect of pH on the PD of MB was also evaluated as various discharges of their effluents at different pH. Figure 5a shows the effect of the pH of the medium on the photodegradation efficiency of MB dye, keeping the irradiation time (1 min) and initial dye concentration (15 ppm) constant. It was observed that the efficiency of photodegradation of MB is much higher in the basic medium than in the acidic medium. It might be because, in the basic medium, the photocatalysts tend to acquire a negative charge that results in increased adsorption of positively charged MB (cationic) dyes because of the rising electrostatic attraction [66]. It is revealed that from an acidic medium to pH 8, the degradation efficiency is almost constant and increases enormously at pH 9. The results confirmed that the calcined rust NP degradation was enhanced from 54% at pH 3 to 95% at pH 9. Similarly, the effect of photocatalyst dosage and initial dye concentration was also evaluated, and the results are represented in Figure 5b,c. Figure 5b represents that photodegradation efficiency increases rapidly by increasing calcined rust NP dosages up to a specific limit (optimum amount) and then levelling off beyond that limit. The results verified that the PD rapidly increased by a 0.02 g photocatalyst dose and a maximum of 82.5% MB dye degraded in 1 min. However, further dose increments showed very little enhancement in the PD performance of the photocatalyst. At a 0.035 g catalyst dose, the PD efficiency increases to ~93% within the same time. The levelling in PD with increasing catalyst dosage might be the increase in solution opacity, which decreases the penetration of the photon flux and ultimately decreases the photocatalytic degradation efficiency [17]. The effect of the initial MB dye concentration is consolidated in Figure 5c, displaying that a maximum PD is achieved at a lower concentration. According to the results, a maximum PD of 92% occurs at an initial concentration of 5 ppm, which frequently decreases to 72.9% by increasing the dye concentration to 25 ppm. Such decreases in PD with the increasing dye concentration are due to the more adsorption of dye molecules on the catalyst surface and occupying its active sites. The adsorb dye molecules absorb a significant amount of light rather than a catalyst, decreasing the generation of hydroxyl radicals and reducing the photocatalytic efficiency [67].

**Figure 6:**
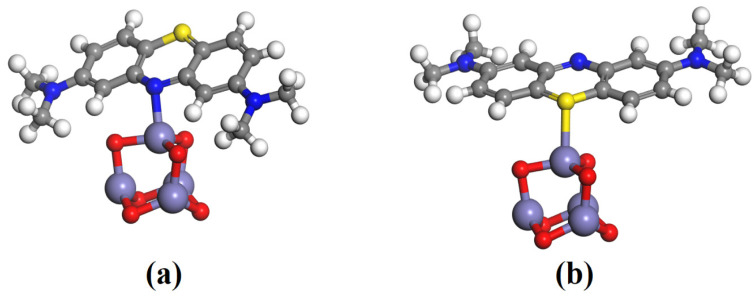
Optimized geometry of the MB adsorption over the Fe_2_O_3_ NPs from the nitrogen site (**a**) and sulfur site (**b**). The white, grey, dark blue, red, yellow, and light blue colors represent hydrogen, carbon, nitrogen, oxygen, sulfur, and iron atom.

Furthermore, we performed the density functional theory simulations to evaluate the degradation mechanism of calcined rust NPs using DMol3 code implanted in Material Studio software [43,44]. Our experimental results demonstrated that the calcinated rust particles are predominantly composed of α-Fe_2_O_3_, which is also mainly responsible for most photocatalytic activities, as suggested by the literature [34,68]. Therefore, we modeled a small cluster of Fe_2_O_3_ NPs as (Fe_2_O_3_)_2_ for simulation. The chemical interactions of the dye molecule with the catalyst surface are very important for degrading a dye by a catalyst. Thus, to check the chemical interactions of MB dye with the calcined rust NPs, we constructed two different complexes of MB and Fe_2_O_3,_ i.e., the MB@Fe_2_O_3_-N(a) and MB@Fe_2_O_3_-S(b) complexes, as shown in Figure 6. N and S represent the interaction of MB with the catalyst surface from the nitrogen site (N), as shown in Figure 6a, and from the sulfur site (S) as shown in Figure 6b). The full geometry optimization reveals that the dye molecule strongly interacted with the Fe_2_O_3_ NPs from both the N and S sites. The bond distance noticed for the N–Fe bond is 1.87 Å and the S–Fe bond is 2.13 Å, respectively. The adsorption energy values noticed for MB@Fe_2_O_3_-N complex is −24.21 kcal/mol, while, for MB@Fe_2_O_3_-S complex, it is −19.47 kcal/mol. The larger adsorption energy values indicate the chemical adsorption of MB over the catalyst surface. This is further justified by the Hirshfeld charge density analysis, which shows a larger charge transfer from the N and S atom to the catalyst surface i.e., −0.31 e and −0.27 e, which resulted in chemical bond formation between the dye molecule and the catalyst. Thus, the chemisorption nature of MB over the catalyst surface reveals that the dye molecule can easily degrade over the catalyst surface.

### 3.4. Photoelectrochemical Water Oxidation

**Figure 7:**
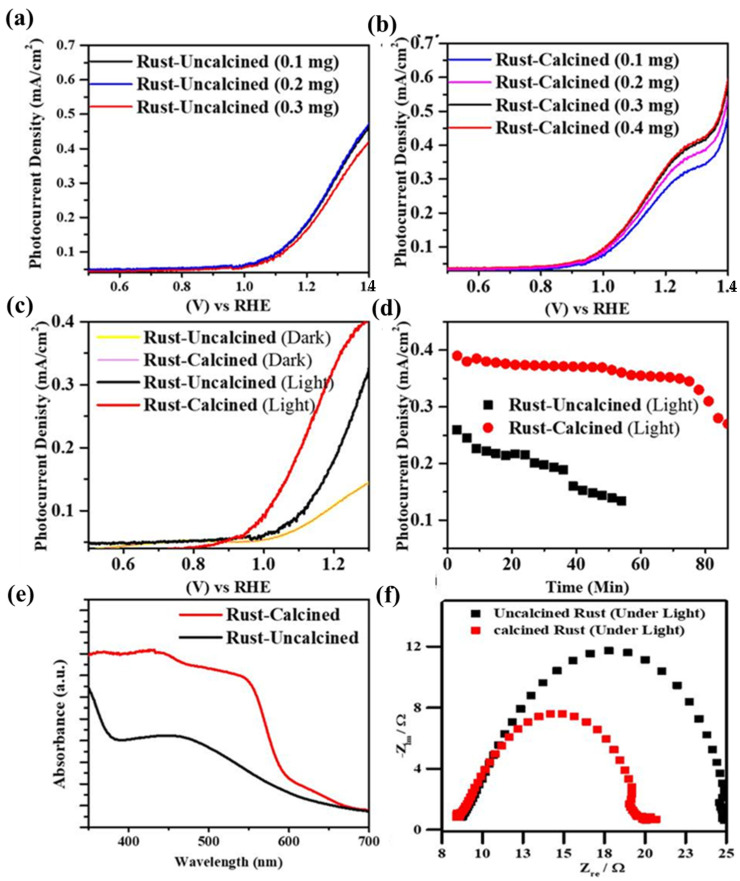
Photoelectrochemical measurements of calcined and uncalcined rust NPs: (**a**,**b**) the catalytic dose–effect under visible light, (**c**) photoelectrochemical linear sweep voltammograms, (**d**) chronoamperometric (I-t) stability curve, (**e**) the UV/Vis-DRS absorption spectra, and (**f**) the EIS Nyquist plots.

The production of H_2_ attracts attention because it is essential for fuel and chemical reactions [2,69]. H_2_ is a sustainable and green form of energy with high energy density, abundance, low cost, and environmentally friendly nature, which makes it an ideal alternative energy candidate to fossil fuels [70]. Both calcined and uncalcined rust were also utilized for water splitting. The PEC performance of the calcined and uncalcined rust NP photoelectrodes was explored via chronoamperometry and linear sweep voltammetry (LSV). Figure 7a,b indicate the dosing effect on the overall water oxidation for calcined and uncalcined photoanodes. It can be seen that increasing the catalyst dose from 0.1 to 0.3 mg/50mL ethanol has a nonsignificant effect in the case of the uncalcined samples. However, the calcined samples indicate enhancement in the activity until 0.3 mg/50mL ethanol; after which, saturation took place, and no further photocurrent density enhancement was observed. Figure 7c displays the LSV measurements under regular solar illumination. For both the samples, the photocurrent remained significantly lower under dark, while, under the simulated light, the photocurrent densities increased enormously beyond 0.7 V vs. RHE with the voltage sweep, as indicated by the I–V spectra. The spectra represent that both materials are photoactive, but the photocurrent density of calcined rust NPs (0.42 mA cm^−2^) is much higher than uncalcined rust NPs (0.34 mA cm^−2^), which can be attributed to the good crystallinity and porosity of the calcined sample. A significant dark current appeared above 1.23 V vs. RHE for the calcined rust NP photoelectrodes, which can be attributed to electrochemical processes [13]. The photocurrent densities observed at the thermodynamic potential (1.23 V vs. RHE) of the water oxidation reaction were ~0.40 and ~0.32 mA/cm^2^ for calcined and uncalcined rust, respectively.

Moreover, it is important to state that the onset potential of calcined rust NPs shifts cathodically to 0.96 V vs. RHE from 1.07 V vs. RHE (for uncalcined samples). The photodegradation efficiency and photostability (good cathodic onset potential shift) of calcined rust NPs indicate an excellent photoelectrocatalytic nature of rust NPs for future energy and environment applications. Figure 7d shows the long-term I–t photostability curve obtained from photoanodes under light with an illumination period of 80 min under calibrated simulated 1 SUN power. The results revealed that the stability curve of calcined rust NPs decreased insignificantly and showed small photo corrosion decay, hence offering considerable resilience and stability during the irradiation period. Similarly, the stability curve of uncalcined rust NPs significantly decreased with more considerable photo corrosion decay, which could be attributed to the photo corrosion of this material due to structural faults and a high charge recombination rate. The uncalcined rust NPs lost their PEC activity entirely after 57 min due to uncontrolled photo decay.

The optoelectrical characteristics are useful to reflect the photoactivity of the samples. For this purpose, we performed a UV/Vis diffuse reflectance analysis of rust. The samples were measured using a quartz cell, with a wavelength range from 200 to 800 nm at a scan rate of 50 nm/min. As indicated in Figure 7e, the calcined samples show superb enhancement in visible light absorption. The onset value of absorption in the range of 580–600 nm for the calcined sample indicates that the bandgap value is well in the order of the visible light region and suitable for PEC water splitting application. The samples are expected to show improved photocatalytic water splitting and dye degradation performance, which is the case as discussed above. To further support the claim, we also measured the samples’ resistivity and surface transport behavior via electronic impedance spectroscopy (EIS) at 0.2 V vs. SCE under AM 1.5 G irradiation, as shown in Figure 7f. Based on the semicircle diameter of the Nyquist plots, one can realize the interfacial charge transport performance [34,71]. The larger the semicircle, the lower the transportation and vice versa. By comparing the circles, it can be concluded that the calcined rust sample has more interfacial transport than the uncalcined. This means that the generated electrons/holes are facilely transferred in the case of calcined rust NPs, hence minimizing the charge recombination and enhancing the overall photo and photoelectrocatalytic activity. A comparison table (Table 2) is also provided, showing some existing hematite-based materials with their photocurrent densities and photo abilities. It is evident from the comparison that calcined rust NPs possess encouraging PEC outcomes, and if the impurities in the rust are controlled, the outcome can be further enhanced. 

## 4. Conclusions

The calcination of rust is an economical and straightforward way for efficient Fe_2_O_3_ NPs photocatalysts preparation. Calcined rust NPs are a novel, economical, and efficient photocatalyst for the PD of MB dye in an aqueous medium and water oxidation under visible light. Due to its high-temperature calcination, the enhanced photocatalytic efficiency of calcined to uncalcined rust NPs makes the materials porous and performs efficient dye adsorption, followed by PD. The calcined rust NPs are highly sustainable and can be used several times. The enhanced PD in the basic medium is due to more hydroxyl radical generation in the alkaline medium. The efficiency of PD increases incrementally with the photocatalyst dosage and a decrease in the initial dye concentration. The computational simulations demonstrate that the dye molecule chemically adsorbed over the catalyst surface by making stronger intermolecular N–Fe and S–Fe bonds and larger adsorption energy, i.e., -24.21 and -19.47 kcal/mol, indicating that the dye can easily degrade over the catalyst surface. The enhanced PEC water oxidation activity of calcined rust NPs might be due to its porous structure via high-temperature calcination, which facilitates light absorption and provides a sufficient pathway for carrier transport, suppressing charge recombination.

## Figures and Tables

**Table 2:** Photocurrent densities and photostability of some reported hematite-based photoanodes.

S/No	Materials	Photocurrent Density@1.23 vs. RHE	Photostability Density@1.23 vs. RHE	Ref.
1	α-Fe_2_O_3_/TiO_2_	1.05 mA/cm^2^	2500 s	[34]
	Ag/α-Fe_2_O_3_/TiO_2_	2.59 mA/cm^2^	3600 s	
2	Fe_2_O_3_	1.55 mA/cm^2^	---	[72]
	FeOOH/Fe_2_O	2.40 mA/cm^2^	5 h	
3	Ta:Fe_2_O_3_@Fe_2_O_3_	2.45 mA/cm^2^	5 h	[73]
	NiFe(OH)x/Ta:Fe_2_O_3_@Fe_2_O_3_	3.22 mA/cm^2^	5 h	
4	Fe_2_O_3_	0.12 mA/cm^2^	2 h	[74]
	Fe_2_O_3_/Fe_2_TiO_5_	0.90 mA/cm^2^	2 h	
	Fe_2_O_3_/Fe_2_TiO_5_/CoFe-PBA	1.25 mA/cm^2^	2 h	
5	Uncalcined rust NPs	0.34 mA cm^−2^	20 min	**This work** **This work**
	Calcined rust NPs	0.42 mA cm^−2^	70 min	

## Data Availability

Not applicable.
